# Comparison of hand hygiene opportunities (HHOS) between a us study and in acute care facilities in three other countries

**DOI:** 10.1186/2047-2994-4-S1-P299

**Published:** 2015-06-16

**Authors:** M-L McLaws, J Hines, C Kilpatrick, J Storr, A Voss, C Leroy, D Limbert

**Affiliations:** 1 School of Public Health and Community Medicine, UNSW Medicine, Sydney, Australia; 2 Research & Development, Deb Group Ltd, Denby, UK; 3 S3 Global, Glasgow, UK; 4 Department of Medical Micriobiology and Infectious Diseases, Canisius-Wilhelmina Hospital, and Radboud University Medical Centre, Nijmegen, Netherlands; 5 Deb Australia, Moorebank, Australia

## Introduction

There is a lack of published data on HHOs/patient/day across countries. While HH compliance rates are often published based on observations by auditors, some recognised disadvantages are training required, valuable hours taken to collect representative samples and the Hawthorne effect. Emerging technologies have potential to improve data reliability, timeliness and density. A key challenge is to establish an accepted “denominator” (HHOs) in the compliance equation.

## Objectives

Our aim was to establish the average HHOs/patient/day in a variety of acute care facilities in Australia, Netherlands and United Kingdom based on the WHO 5 Moments for HH recommendations.

## Methods

Australia: 24-hr expert observations of HHOs were made in 2 wards for 7 days. HHOs/patient/day were averaged for both wards, tested for difference and aggregated. Aggregated average HHOs/patient/day were adjusted for care level using patient:nurse ratio and weighted for auditor bias.

Netherlands: Case patients were followed for 7 days to directly observe HHOs/patient/day. Data were tested for difference and averaged.

UK: Commonly performed care scenarios, “virtual patient/HCW observations”, were gathered from a range of wards in 3 hospitals and aggregated into typical care-days representative of patient type by care level to generate HHOs/patient/day. Selected expert observations were completed to validate results.

Average HHOs established from a USA study were then compared against the results from the 3 countries.

## Results

In all 3 countries average HHOs/patient/day were in the range 50-85. Patient:nurse ratios in all 3 countries fell in the range 3-5. All results compared closely with the USA study findings.

## Conclusion

Our work indicates that in acute care facilities in the 3 countries studied and USA, HHOs/patient/day is similar and driven by the prevailing similar patient:nurse ratio as a universal indicator of patient care level. This novel information provides valuable insight and allows emerging technologies that use HHOs as a denominator in a compliance equation to be considered for use in different developed countries where facilities and practices are similar.

## Disclosure of interest

None declared.

